# High Prevalence of Antimicrobial Resistance Among Gram-Negative Isolated Bacilli in Intensive Care Units at a Tertiary-Care Hospital in Yucatán Mexico

**DOI:** 10.3390/medicina55090588

**Published:** 2019-09-13

**Authors:** Andrés H. Uc-Cachón, Carlos Gracida-Osorno, Iván G. Luna-Chi, Jonathan G. Jiménez-Guillermo, Gloria M. Molina-Salinas

**Affiliations:** 1 Medical Research Unit-Yucatán, Medical Unity of High Specialty, Specialty Hospital 1, Mexican Social Security Institute, IMSS, Merida, Yucatán 97150, Mexico; 2 Internal Medicine Service, Regional General Hospital 1, Delegation Yucatán, Mexican Social Security Institute, IMSS, Merida, Yucatan 97150, Mexico; 3 Clinical Pathology Laboratory, Medical Unity of High Specialty, Specialty Hospital 1, Mexican Social Security Institute, IMSS, Merida, Yucatán 97150, Mexico; 4 Faculty of Medicine, Juárez Autonomous University of Tabasco, Villahermosa, Tabasco 86040, Mexico

**Keywords:** antimicrobial resistance, Gram-negative bacilli, enterobacteriaceae, non-fermenting Gram-negative bacilli, ESBL, MDR, HRMO

## Abstract

*Background and Objectives*: Antimicrobial resistance (AMR) is increasing worldwide and imposes significant life-threatening risks to several different populations, especially to those in intensive care units (ICU). The most commonly isolated organisms in ICU comprise gram-negative bacilli (GNB), and these represent a leading cause of serious infections. This study was conducted to describe the prevalence of resistance in GNB isolated from patients in adults, pediatric, and neonatal ICU in a tertiary-care hospital in Mérida, Mexico. *Materials and Methods*: A retrospective study was done on samples collected in Neonatal (NICU), Pediatric (PICU) and Adult (AICU) ICU of Unidad Médica de Alta Especialidad, Instituto Mexicano del Seguro Social in Mérida, México. The identification of isolates and antimicrobial susceptibility testing were performed using an automated system. *Results*: A total of 517 GNB strains were isolated. The most common positive culture was bronchial secretions. *Pseudomonas aeruginosa* was the prevalent pathogen in NICU and PICU, whereas *Escherichia coli* was common in the AICU. Overall, GNB exhibited a high resistance rates for Ampicillin (95.85%), Cefuroxime (84.17%), Piperacillin (82.93%), Cefotaxime (78.07%), Ceftriaxone (77.41%), Aztreonam (75.23%), Cefazolin (75.00%), and Ceftazidime (73.19%). There are significant differences in the resistance rates of GNB from different ICUs for penicillins, cephalosporins, carbapenems and fluoroquinolones drugs. *Escherichia coli* (multidrug-resistant [MDR] = 91.57%, highly resistant microorganisms [HRMO] = 90.36%) and *Acinetobacter baumannii* (MDR = 86.79%, HRMO = 83.02%) exhibited the highest percentage of MDR and HRMO profiles. The prevalence of the extended-spectrum beta-lactamases (ESBL)-producing isolates was 83.13% in *E. coli*, 78.84% in *Klebsiella pneumoniae*, and 66.67% in *Proteus mirabilis*, respectively. *Conclusions*: The high resistance rates to drugs were exhibited by our GNB isolates. Continuous surveillance and control of the use of antimicrobials are urgently needed to reduce the emergence and spreading of MDR, HRMO, and/or ESBL-producing bacilli.

## 1. Introduction

According to the World Health Organization (WHO), antimicrobial resistance (AMR) is a threat to the prevention of and therapy against infectious diseases. It is a global problem caused mainly by poor administration and inadequate therapy, and the use of antimicrobials in an abusive manner without medical supervision. AMR leads to longer hospital stays, higher medical costs, and increased mortality [1]. The cost annual for AMR in the U.S. healthcare system has been estimated as ranging from US $21–$34 billion dollars, accompanied by more than 8 million additional days in the hospital [2]. Additionally, in the U.S., more than 2 million persons were infected with resistant bacteria and 23,000 died as a result of these infections [3]. In countries of the European Union, the costs for infections caused by resistant bacteria exceeded 1.6 million €, with 2.5 million additional days of hospital stays, and these infections caused 25,000 deaths [4].

AMR increases by 700,000, the number of deaths annually, and for 2050, the number is estimated to be 10,000,000, overtaking that of cancer [5]. AMR is increasing global [6]. In recent years, a great number of resistant strains have emerged in different pathogens, and multidrug-resistant bacteria (MDR) gram-negative pathogens are also becoming increasingly prevalent in the community [2]. The specific definition of MDR is labelled as such because of their in vitro resistance to three or more antimicrobial classes of drugs [7]. In a recent report, the WHO published a list of antibiotic-resistant priority pathogens, in which bacteria with critical priority included *Acinetobacter* spp. and *Pseudomonas* spp. with carbapenem resistance, and Enterobacteriaceae family members that produced the extended-spectrum beta-lactamases (ESBL) and carbapenemases [8].

The mechanisms of drug resistance fall into several broad categories including drug degradation/alteration (such as ESBL, aminoglycoside-modifying enzymes, or Chloramphenicol acetyltransferases); the modification of drug binding sites/target; the changes in cell permeability and efflux pump expression, resulting in reduced intracellular drug accumulation [9].

Recent studies of the prevalence of AMR have shown a high increase in resistant infections, especially in intensive care unit (ICU) areas [10,11,12,13]. The patients in ICU are vulnerable as they are exposed to different invasive procedures, such as intubation, mechanical ventilation, and vascular access. In addition, many of the drugs employed can give rise to inhibition of their immune response [14]. The different studies have reported that the most commonly isolated organisms in patients in ICU comprise gram-negative bacilli (GNB), mainly Enterobacteriaceae species, and non-fermenting gram-negative bacilli (NF-GNB), such as *Pseudomonas* and *Acinetobacter* species [13,15,16]. GNB were responsible for 45–70% of ventilator-associated pneumonias, for 20–30% of vascular pathway infections (catheter), and for commonly caused infections in surgical wounds and in the urinary tract that led to sepsis [17]. In addition, in the last several years, the occurrence of highly resistant microorganisms (HRMO) is a major threat to patients in ICU, leading to worse outcomes, the need for isolation measures, and the demand for second-line or rescue antibiotics [18].

AMR varies from country to country and from to region to region depending on policies for antimicrobial use and the knowledge of its prevalence, and local resistance patterns may aid in better management of patients and the development of better antimicrobial stewardship programs. The aim of this study was to determine the prevalence of AMR in the clinical isolates of GNB from adult, pediatric, and neonatal patients in these ICU at a tertiary-care hospital in Mexico.

## 2. Materials and Methods

### 2.1. Site and Period of Study

This retrospective study involved all bacteriological samples collected in neonatal (NICU), pediatric (PICU), and adult (AICU) patients of these ICU at the Unidad Médica de Alta Especialidad (UMAE), Instituto Mexicano del Seguro Social (IMSS), in Mérida, state of Yucatán, Mexico. The protocol was approved by the Local Committees on Health Research 3203 and Ethics 32038 of the UMAE with registration number R-2018-3203-021. The data of the culture reports during the period from 1 August, 2016 to 31 December, 2018 were analyzed.

### 2.2. Microbiology

#### 2.2.1. Culture and Identification

The clinical samples were collected appropriately at the bed-side and then transported to the Microbiology Laboratory. For this purpose, this study employed sterile bottles, sterile dry cotton swabs, versaTREK REDOX bottles (Thermo Fisher Scientific, Inc., Waltham, MA, USA), and sterile tubes with a Brain Heart Infusion medium depending on the sample type. Each specimen was plated onto MacConkey, Blood, Mannitol Salt, and Chocolate agars (Becton Dickinson & Co., Franklin Lakes, NJ, USA). The inoculated agars were incubated aerobically at 36 °C for 24 h. The bacterial species were classified by morphological-colony characteristics, Gram staining, and by routine rapid tests, such as catalase and oxidase. The organism identification was carried out using the automated MICROSCAN WalkAway^®^ system (Beckman Coulter, Inc., Brea, CA, USA). MICROSCAN panels utilized modified conventional and chromogenic tests for the identification of Enterobacteriaceae species and NF-GNB. The identification was based on the detection of pH changes, substrate utilization, biochemical overlays, and growth in the presence of antimicrobial agents after 12–48 h of incubation at 35 °C [19]. 

#### 2.2.2. Test Drugs

A total of eight types of drugs were tested for all microorganisms. The drugs included the following: (a) Penicillins, such as Ampicillin and Piperacillin; (b) penicillin-beta-lactamase inhibitor combination, such as Ampicillin/Sulbactam, Piperacillin/Tazobactam, and Ticarcillin/Clavulanic Acid; (c) cephalosporins, such as Cefepime, Cefotaxime, Cefuroxime, Cefazolin, Cefotetan, Ceftriaxone, and Ceftazidime; (d) aminoglycosides, such as Amikacin, Gentamicin, and Tobramycin; (e) carbapenems, such as Imipenem and Meropenem; (f) fluoroquinolones, such as Ciprofloxacin, Levofloxacin, and Moxifloxacin; (g) trimethoprims, such as Trimethoprim/Sulfamethoxazole, and (h) monobactams, such as Aztreonam.

#### 2.2.3. Antimicrobial Susceptibility Testing

Antimicrobial susceptibility was carried out with the help of the MICROSCAN WalkAway^®^ system using Negative/urine combo 55 and Negative combo 68 panels. The tests carried out on MICROSCAN were miniaturizations of broth microdilution susceptibility tests that were dehydrated. The various antimicrobial agents were diluted in Mueller-Hilton broth supplemented with calcium and magnesium to concentrations bridging the range-of-clinical-interest after inoculation and rehydration with a standardized suspension of the organism and incubation at 35 °C for a minimum of 16 h [20,21]. The minimal inhibitory concentration (MIC) for the test organism was determined by observing the lowest antimicrobial concentration exhibiting growth inhibition, and the quality antimicrobial susceptibility pattern (S: Susceptible, I: Intermediate, and R: Resistant) was determined per Clinical and Laboratory Standards Institute (CLSI) guidelines [20].

#### 2.2.4. Detection of Extended-Spectrum Beta-Lactamases (ESBL)

The screening for ESBL detection was performed on the MICROSCAN WalkAway^®^ system employing antimicrobial susceptibility tests using Ceftazidime and Cefotaxime, with or without Clavulanic Acid [20].

### 2.3. Statistical Analysis

The data were entered and analyzed using Microsoft Excel. The distribution of the specimens and bacterial isolates was expressed as percentages. AMR, ESBL-producing, MDR, and HRMO profiles of clinical isolates were analyzed and expressed as percentages.

## 3. Results

### 3.1. Bacterial Isolates

**Figure 1:**
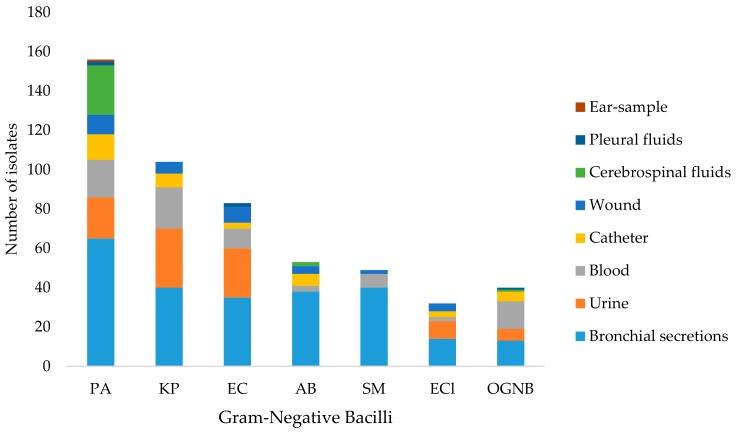
Distribution of Gram-negative bacilli (GNB) recovered from clinical samples of patients in intensive care units (ICU). PA: *P. aeruginosa*, KP: *K. pneumoniae*, EC: *E. coli*, AB: *A. baumannii*; SM: *Stenotrophomonas maltophilia*; ECl: *Enterobacter cloacae*; OGNB: Other GNB. OGNB included *P. mirabilis* (*n* = 9), *Serratia marcescens* (*n* = 9), *Burkholderia* spp. (*n* = 9), *A. lwoffi* (*n* = 8), and *Klebsiella oxytoca* (*n* = 5).

A total of 2,711 clinical samples were analyzed and 517 GNB strains were isolated. The distribution of GNB recovered from various clinical specimens of ICU patients is summarized in Figure 1 and Table 1. The majority of isolates were recovered from the clinical samples of bronchial secretions (*n* = 245), urinary samples (*n* = 91), and blood samples (*n* = 76). *Pseudomonas aeruginosa* was the predominant isolate (*n* = 156, 30.17%), followed by *K. pneumoniae* (*n* = 104, 20.12%), and *E. coli* (*n* = 83, 16.05%).

**Figure 2:**
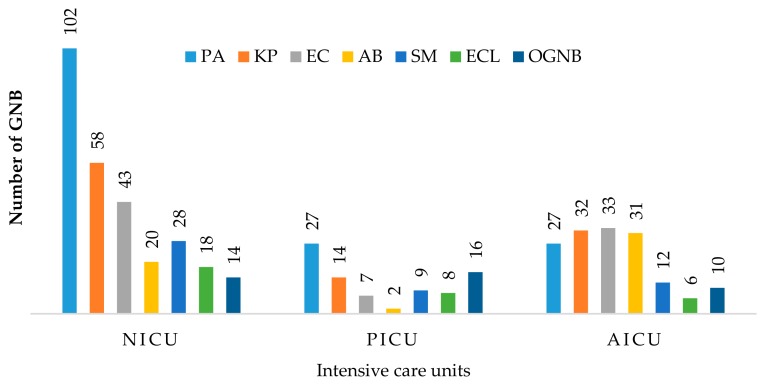
Frequency of GNB among three intensive care units (Neonatal, NICU, Pediatric, PICU, and Adult, AICU) PA: *P. aeruginosa*, KP: *K. pneumoniae*, EC: *E. coli*, AB: *A. baumannii*; SM: *S. maltophilia*; ECl: *E. cloacae*; OGNB: Other GNB. OGNB including *P. mirabilis* (*n* = 9), *S. marcescens* (*n* = 9), *Burkholderia* spp. (*n* = 9), *A. lwoffi* (*n* = 8). and *K. oxytoca* (*n* = 5).

According to the distribution of isolates by ICU type, *P. aeruginosa* and *K. pneumoniae* were the most prevalent pathogens in NICU and PICU. Moreover, *E. coli*, *K. pneumoniae*, *A. baumannii*, and *P. aeruginosa* were the most frequent isolates from the AICU (Figure 2).

### 3.2. AMR of the Clinical Isolates

The drug resistance for GNB clinical isolates is depicted in Table 2. Overall, the highest resistant rate was found for Ampicillin (95.85%), followed by Cefuroxime (84.17%), Piperacillin (82.93%), Cefotaxime (78.07%), Ceftriaxone (77.41%), Aztreonam (75.23%), Cefazolin (75.00%), and Ceftazidime (73.19%). The lowest resistance rate was observed for Cefotetan (10.96%), and carbapenems (27.42–33.41%).

The enterobacterial isolates of *K. pneumoniae*, *E. coli*, and *E. cloacae* revealed high resistance rates to penicillins (97.12–75.00%), which was reversed by the addition of an ESBL inhibitor, such as Sulbactam and Tazobactam, cephalosporins (100–59.37%) except for Cefotetan, Aztreonam (88.89–87.50%), and Tobramycin (79.01–48.39%). Additionally, clinical isolates of *E. coli* displayed high resistance rates to fluoroquinolones (78.31–70.00%).

The clinical isolates of *A. baumannii* exhibited high resistance rates to third- and fourth-generation cephalosporins (81.13–67.92%), Ciprofloxacin (79.25%), Gentamicin (84.91%), and Trimethoprim/Sulfamethoxazole (77.36%). The clinical isolates of *P. aeruginosa* revealed high resistance rates to Piperacillin (70.59%) and Imipenem (69.68%). Other GNB (OGNB) that included *P. mirabilis*, *S. marcescens*, *Burkholderia* spp., *A. lwoffi*, and *K. oxytoca* revealed high resistance rates to Ampicillin (100%), Cefazolin (80.00%), Cefuroxime (81.82%), Ceftazidime (66.67%), and Aztreonam (66.67%).

### 3.3. MDR, HRMO Profiles, and ESBL-Producing

**Figure 3:**
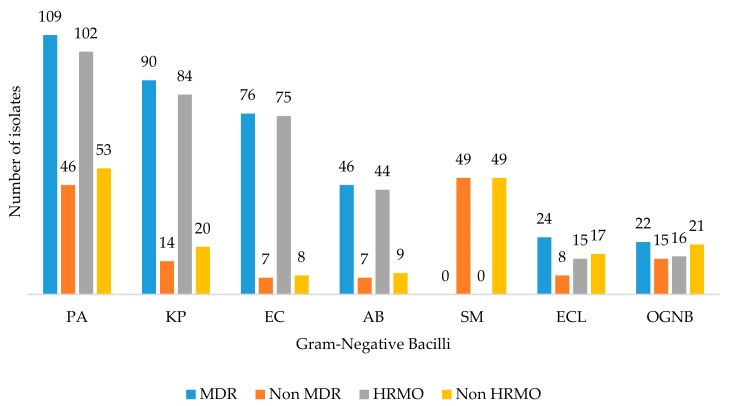
Frequency of multidrug-resistant (MDR) and highly resistant microorganisms (HRMO) GNB recovered from patients in the three ICU. PA: *P. aeruginosa*; KP: *K. pneumoniae*; EC: *E. coli*; AB: *A. baumannii*; SM: *S. maltophilia*; ECl: *E. cloacae*; OGNB: Other GNB. OGNB include *P. mirabilis* (*n* = 9), *S. marcescens* (*n* = 9), *Burkholderia* spp. (*n* = 9), *A. lwoffi* (*n* = 8). and *K. oxytoca* (*n* = 5).

The frequency of MDR and HRMO rates is illustrated in Figure 3. *Escherichia coli* (MDR = 91.57%, HRMO = 90.36%), *A. baumannii* (MDR = 86.79%, HRMO = 83.02%), and *K. pneumoniae* (MDR = 83.65%, HRMO = 80.77%) exhibited the highest percentage of MDR and HRMO profiles in clinical isolates and, overall, 71.65% and 65.50% of total GNB exhibited MDR and HRMO profiles, respectively.

Moreover, the prevalence of the ESBL-producing was 83.13% in *E. coli*, 78.84% in *K. pneumonia*, and 66.67% in *P. mirabilis*.

## 4. Discussion

In hospitals ICU, due to their characteristics, allow for concentration factors that lead to AMR. These factors include the frequent use of broad-spectrum antimicrobial drugs, invasive procedures and devices, patients with a high frequency of comorbidity, and prolonged hospital stays, among others [13]. The present study investigated the spectrum of the GNB clinical isolates of patients hospitalized in ICU and their AMR pattern in a tertiary-care level hospital in the state of Yucatán, Mexico.

### 4.1. Bacterial Isolates

A total of 517 GNB were isolated from 2,711 clinical samples analyzed. In this study, the most common organism isolated from patients in an ICU was *P. aeruginosa* (*n* = 156; 30.41%). Our findings were in agreement with another study conducted in an ICU of a tertiary-care hospital located in India [11].

*Klebsiella pneumoniae* (*n* = 104; 20.27%) and *E. coli* (*n* = 83; 16.17%) were the most common enterobacterial clinical isolates, similar to those previously reported by Moolchandani et al. [11], in clinical isolates of patients in an ICU in India.

In the case of NF-GNB, the most prevalent species isolated were *P. aeruginosa* (*n* = 156; 30.41%) and *A. baumannii* (*n* = 53; 10.33%). Similar data were reported in studies conducted in three hospitals in Mexico. The most prevalent species found was *P. aeruginosa* (24%) followed by *A. baumannii* (12.5%) [22].

The most common culture-positive clinical samples received during the present study were bronchial secretions (40.64%). Similar findings were obtained by Moolchandani et al. [11] in a study conducted in an ICU of a tertiary-care level hospital in Southern India [10]. This is probably due to ventilator-associated pneumonia, which is one of principal infections in ICU and which occurs 48–72 h after the initiation of mechanical ventilation [23]. Interestingly, the most prevalent GNB isolate for bronchial secretions was the NF-GNB *P. aeruginosa* (42.30%). In the ICU of a hospital in Southern India, NF-GNB *Acinetobacter* spp. (36.00%) and *Pseudomonas* spp. (27.6%) were those most prevalent in bronchial secretions. This suggests that this is due to an increase in the colonization of NF-GNB in the respiratory tract of patients on prolonged mechanical ventilation [11].

### 4.2. AMR of the Clinical Isolates 

AMR in hospitals is driven by the failures of hospitals in terms of hygiene, selective pressures created by the overuse of antimicrobial drugs, and mobile genetic elements that can encode mechanisms of bacterial resistance [24]. The increasing drug resistance of *K. pneumoniae* and *E. coli* in the last few decades has been a problem worldwide [25], and these GNB were the most prevalent among our enterobacterial clinical isolates. The clinical isolates of *K. pneumoniae* and *E. coli* exhibited high resistance rates to various groups of antimicrobial drugs such as penicillins (100–80.00%), cephalosporins (87.95–60.97%), Aztreonam (88.89–87.84%), and Tobramycin (79.01–61.76%). The high resistance rates to penicillins and cephalosporins were also detected in clinical isolates of patients in ICU from Vietnam [13], India [11], and Nepal [10]. Our results of drug resistance rates to Aztreonam and Tobramycin were higher than those reported for clinical isolates of patients in a hospital ICU in Saudi Arabia, where the resistance rates reported for these antimicrobials were within the range of 65.2–30.6% [26].

In addition, the clinical isolates of *E. coli* exhibited high resistance rates to all quinolone drugs evaluated (78.31–70.00%). In a Latin-American country such as Venezuela, since 2011, the resistance rates of *E. coli* to quinolones reached 46.51% and, for 2012, the tendency for Levofloxacin and Moxifloxacin was of 100% [27]. On the other hand, in a study conducted at three hospitals in Mexico, the resistance rates of *E. coli* to Ciprofloxacin was 55.56% [22]. Regarding another part of the world, such as ICU in Nepal and India, the number of strains resistant to Ciprofloxacin is greater, up to 90.1%, and to Levofloxacin, 77.4% [10,11].

NF-GNB have high intrinsic resistance to antimicrobial drugs due to the low outer-membrane permeability, together with secondary resistance mechanisms, such as inducible cephalosporinase or antibiotic efflux pumps. Therefore, the number of antimicrobial drugs is reduced [28]. Among the NF-GNB isolates of our patients, *A. baumannii* exhibited high resistance rates to a large number of antimicrobial drugs tested, such as cephalosporins, Ciprofloxacin, Gentamicin, and Trimethoprim/Sulfamethoxazole. Cephalosporin drugs play an important role in antimicrobial treatment. The *A. baumannii* isolates demonstrated high resistance rates, between 79.25 and 75.47%. The high resistance rates to Cephalosporin were also detected in a multicenter study conducted in three hospitals in Mexico. The resistance rates reported for these groups of antimicrobials was in the range of 100–55.56% [22]. In an ICU from hospitals located in Argentina, Chile, and Venezuela, in terms of *A. baumannii*, the number of resistant isolates was above 60% between the years 2004 and 2010 [27]. On the other hand, recent reports in hospitals in countries in Africa and South Asia revealed higher results than this study: Cephalosporin resistance rates in clinical isolates from patients in ICU of hospitals in Morocco and Vietnam ranged from 94.66–95.8% and from 93–95%, respectively [12,13]. In a hospital in Nepal, entire isolates were resistant to Ceftazidime and Cefepime [10]. In the case of Ciprofloxacin, Gentamicin, and Trimethoprim/Sulfamethoxazole, our results revealed the prevalence of resistance in more than 79.25% of *A. baumannii* clinical isolates. Our resistance results were lower than those reported for the clinical isolates of patients in hospital ICU in India [11], Vietnam [13], and Saudi Arabia [26]. Parajuli et al. [10] suggested that these high resistance levels of *Acinetobacter* spp. may be due to the elevated change of the acquisition of resistant genes and their ability to persist and multiply in a hospital environment [10].

The clinical isolates of *P. aeruginosa* exhibited high resistance rates to Imipenem (69.68%) and Piperacillin (70.59%). The effect of Piperacillin was improved with a β-lactamase inhibitor such as Tazobactam. Our findings on Imipenem are disturbing. *P. aeruginosa* was the most common organism isolated and 69.68% of these clinical isolates were resistant to Imipenem. Imipenem is the best reserve drug employed in the treatment of infections caused by MDR strains. Imipenem-resistant strains are often resistant to other antimicrobial drugs, and the prognosis is worse yet in terms of mortality and morbidity [29]. Imipenem resistance may be mediated by diminished outer membrane permeability, efflux pump over-expression, and carbapenemase enzymes. This later type of resistance is the most important clinically because these enzymes hydrolyze all or nearly all beta-lactams drugs. Carbapenemases are encoded by genes that are horizontally transferable by plasmids or transposons and commonly associated with genes encoding for other resistance determinants [30]. Carbapenemases, such as IMP- (Imipenem-resistant Pseudomonas), GES- (Guiana extended-spectrum beta-lactamase), VIM- (Veron integron-encoded metallo-beta-lactamase), and OXA-type (oxacillin-hydrolyzing), have been reported in clinical isolates of *P. aeruginosa* from patients in a hospital in Mexico [31,32]. On the other hand, our Imipenem resistance rates were higher than those reported for the clinical isolates from patients in ICU of a hospital in Saudi Arabia (38.2%) [26]. Similar findings were reported in isolates from patients in ICU in hospitals in Vietnam (79.3%) [13] and Nepal (62.5%) [10].

### 4.3. MDR, HRMO Profiles, and ESBL-Producing

This study found that 70.96% of GNB isolates demonstrated an MDR profile. This is high compared with that previously reported by Moolchandani et al. [11] in a study conducted in patients of ICU of hospitals in India (55.7%) [11]. Specifically, the clinical isolates of *E. coli* (91.57%), *A. baumannii* (86.79%), and *K. pneumoniae* (83.65%) comprised the GNB species with the greatest number of MDR strains. Our findings were in accordance with those of Moolchandani et al. [11] with respect to *A. baumannii* (82.1%). However, these were higher for *E. coli* (26.3%) and *Klebsiella* spp. (52.4%) [11]. In addition, the prevalence of the MDR profile of clinical isolates from this research was higher than that reported for patients at the Hospital Universitario of Monterrey, Nuevo León, Mexico, where the global prevalence of GNB with an MDR profile was 46.2% and included *A. baumannii* (74%), *E. coli* (40%), *P. aeruginosa* (34%), *K. pneumoniae* (22%), *E. cloacae* (9%), and *Serratia* spp. (7%) [33]. The prevalence of MDR-GNB is high even in developed countries. During 2013 in the U.S., 63% of isolates of *A. baumannii* presented an MDR profile, and it has been estimated that 7,300 infections were caused by these isolates [34].

HRMO are microorganisms that 1) are known to cause disease, 2) have acquired an AMR pattern that hampers therapy, and 3) have the potential to spread if, in addition to standard precautions, no transmission-based precautions are taken [35]. Kluytmans-VandenBergh et al. [35] defined HRMO as (1) Enterobacteriaceae that are ESBL-producing and/or resistant to carbapenems and/or resistant to fluoroquinolones and aminoglycosides (F and A), (2) *Acinetobacter* spp. that are resistant to carbapenems and/or resistant to F and A, (3) *S. maltophilia* resistant to Co-trimoxazole, and (4) *P. aeruginosa* resistant to at least three of following antimicrobial or antimicrobial groups: Piperacillin, Ceftazidime, F and A, and/or carbapenems [35,36]. This study demonstrated a high number of clinical isolates with an HRMO profile. A total of 65.50% of GNB exhibited the HRMO profile. The highest percentages of HRMO were recovered in clinical isolates of *E. coli* (90.36%), *A. baumannii* (83.02%), and *K. pneumoniae* (80.77%).

One of type of drug resistance comprises ESBL enzymes in the Enterobacteriaceae family, which permit the bacteria to resist the effect of penicillins and cephalosporins of first-, second-, and third-generation penicillins and cephalphorins, as well as Aztreonam [37]. In this study, 83.13% (69/83) of the clinical isolates of *E. coli*, 78.84% (82/104), of *K. pneumoniae*, and 66.67% (6/9) of *P. mirabilis* were ESBL-producing. The data in this study were higher than those reported in studies conducted in a hospital ICU from Nepal and India. With respect to research developed at a hospital in Nepal, ESBL isolates of *E. coli* and *Klebsiella* spp. exhibited 70.90% and 59.40%, respectively [10]. In case of a hospital in India, the prevalence of ESBL-producing was 47.6% for *E. coli*, 15.4% for *Klebsiella* spp., and 0% for *Proteus* spp. [38]. On the other hand, from 2009–2011, 13.7% and 16.6% of clinical isolates of *E. coli* were ESBL-producing in the U.S. and in European countries, respectively. The ESBL-producing isolates of *Klebsiella* spp. were reported to have undergone an increase in the prevalence of 27.5–42.8% in Europe during the same years [39]. Even so, the data of prevalence of ESBL-producing isolates of *E. coli* (83.13%) were higher than those reported by the SMART (Study for Monitoring Antimicrobial Resistance Trends) group with 41% [40]. Moreover, the SENTRY Antimicrobial Surveillance Program of Mexico reported a prevalence of 33% of ESBL-producing *K. pneumoniae* and at our hospital, 78.84% was found. This study’s data were also higher than those reported for Argentina (60%), Brazil (50%), and Chile (59%) [40]. The research studies on prevalence in Monterrey, N.L., Mexico, informed ESBL-producing in 35.9% of clinical isolates of *K. pneumoniae* and 35.6% of *E. coli* [41]. Morfín-Otero et al. [42] reported lower data for *E. coli* (16.3%) and *K. pneumoniae* (26.9%) in isolates of patients in Guadalajara Jalisco, Mexico [42]. In Mexico, CTX-M-15, and SHV-12 are the most commonly detected ESBL from patients in hospitals. In addition, CTX-M-9, SHV-5, GES-1, GES-19, TEM-1 and TLA-1 have also been found [43,44,45,46]. ESBL-producing in *K. pneumoniae* and *E. coli* isolates support the high level of resistance to Ampicillin (97.12–93.98%), Cephalosporin (91.18–60.97%), and Aztreonam (88.89–87.84%) in this research.

### 4.4. Study Limitations

First, MICROSCAN WalkAway^®^ system are not able to differentiate among the species of the *Burkholderia cepacia* complex. Second, the data of MIC testing in/of clinical isolates were taken from the Microbiology Laboratory reports and sometimes these were incomplete. Third, the identification of MDR and HRMO profiles were based on the number of antimicrobial drug classes that were evaluated in the Microbiology Laboratory.

## 5. Conclusions

It can be concluded from this study that the most commonly isolated GNB were *P. aeruginosa*, *K. pneumoniae*, *E. coli*, and *A. baumannii*. The enterobacterial isolates revealed high resistance rates to Ampicillin and Aztreonam. Additionally, *E. coli* demonstrated high resistance rates to quinolones. Generally speaking, *A. baumannii* isolates were highly resistant to a large number of drugs tested. The clinical isolates of *P. aeruginosa* exhibited high resistance rates to Imipenem, the best reserve drug employed in the treatment of MDR infections. Finally, this study showed a higher rate of MDR and HRMO profiles and ESBL-producing in GNB clinical isolates. This finding herein emphasizes the need for continuous surveillance and rational treatment strategies to reduce the emergence and spreading of MDR- and HMRO-GNB.

## Figures and Tables

**Table 1:** Distribution of GNB recovered from clinical samples of patients in ICU.

Sample	Total Number of Isolates	PA	KP	EC	AB	SM	ECl	OGNB
Bronchial secretions	245	65	40	35	38	40	14	13
Urine	91	21	30	25	0	0	9	6
Blood	76	19	21	10	3	7	2	14
Catheter	37	13	7	3	6	0	3	5
Wound	34	10	6	8	4	2	4	0
Cerebrospinal fluids	28	25	0	0	2	0	0	1
Pleural fluids	5	2	0	2	0	0	0	1
Ear-sample	1	1	0	0	0	0	0	0
Total (%)	**517**	**156 (30.17)**	**104 (20.12)**	**83 (16.05)**	**53 (10.25)**	**49 (9.48)**	**32** **(6.19)**	**40** **(7.74)**

GNB: Gram-Negative Bacilli, ICU: intensive care units, PA: *P. aeruginosa*, KP: *K. pneumoniae*, EC: *E. coli*, AB: *A. baumannii,* SM: *S. maltophilia*, ECl: *E. cloacae,* OGNB: Other GNB, including *P. mirabilis* (*n* = 9), *S. marcescens* (*n* = 9), *Burkholderia* spp. (*n* = 9), *A. lwoffi* (*n* = 8), and *K. oxytoca* (*n* = 5).

**Table 2:** Proportions and percentage of drug-resistant GNB recovered from clinical samples of patients in ICU.

Antimicrobial Drugs	PA	KN	EC	AB	SM	ECl	OGNB	Overall Isolates
Ampicillin		101/104 (97.12)	78/83 (93.98)			30/32 (93.75)	22/22 (100)	231/241 (95.85)
Ampicillin/ Sulbactam		71/104 (68.27)	64/83 (77.11)	28/53 (52.83)		24/32 (75.00)	13/29 (44.83)	200/301 (66.67)
Piperacillin	12/17 (70.59)	30/30 (100)	20/25 (80.00)			6/8 (75.00)	0/2 (0.00)	68/82 (82.93)
Piperacillin/ Tazobactam	71/155 (45.80)	28/103 (27.18)	28/81 (34.57)			8/32 (25.00)	4/22 (18.18)	139/393 (35.36)
Ticarcillin/ Clavulanic acid	26/40 (65.00)	11/28 (39.29)	16/29 (55.17)	16/28 (57.14)	10/28 (35.71)	4/7 (57.14)	2/14 (14.29)	85/174 (48.85)
Cefazolin		25/41 (60.97)	34/411 (82.93)			8/8 (100)	8/10 (80.00)	75/100 (75.00)
Cefotetan		7/62 (11.29)	1/57 (1.75)			6/16 (37.50)	2/11 (18.18)	16/146 (10.96)
Cefuroxime		84/103 (81.55)	73/83 (87.95)			27/32 (84.98)	18/22 (81.82)	202/240 (84.17)
Cefotaxime		84/104 (80.76)	72/83 (86.75)	40/53 (75.47)		22/32 (68.75)	17/29 (58.62)	235/301 (78.07)
Ceftazidime	100/154 (64.93)	84/104 (80.76)	73/83 (87.95)	43/53 (81.13)	28/49 (57.14)	22/32 (68.75)	24/36 (66.67)	374/511 (73.19)
Ceftriaxone		84/104 (80.76)	73/83 (87.95)	36/53 (67.92)		23/32 (71.88)	17/29 (58.62)	233/301 (77.41)
Cefepime	83/155 (55.13)	83/104 (79.80)	71/83 (85.54)	40/53 (75.47)		19/32 (59.37)	17/29 (58.62)	313/456 (68.64)
Aztreonam	24/57 (42.11)	65/74 (87.84)	56/63 (88.89)			14/16 (87.50)	8/12 (66.67)	167/222 (75.23)
Imipenem	108/155 (69.68)	10/104 (9.62)	3/83 (3.61)			7/32 (21.88)	2/15 (13.33)	130/389 (33.41)
Meropenem	84/155 (54.19)	7/104 (6.73)	2/83 (2.41)	30/53 (56.60)		1/32 (3.13)	3/36 (8.33)	127/463 (27.42)
Ciprofloxacin	65/155 (41.93)	32/104 (30.77)	65/83 (78.31)	42/53 (79.25)		16/32 (50.00)	8/29 (27.59)	228/456 (49.34)
Levofloxacin	61/155 (39.35)	16/104 (15.38)	63/83 (75.90)	32/53 (60.38)	3/49 (6.12)	4/32 (12.50)	7/36 (19.44)	186/512 (36.32)
Moxifloxacin		5/28 (17.86)	21/30 (70.00)			1/8 (12.50)	1/9 (11.11)	28/75 (37.33)
Amikacin	90/155 (58.06)	14/104 (13.46)	8/83 (9.64)	31/53 (58.49)		5/32 (15.63)	10/29 (34.48)	158/456 (34.65)
Gentamicin	80/155 (51.61)	48/104 (46.15)	52/83 (62.65)	45/53 (84.91)		9/32 (28.13)	8/29 (27.59)	242/456 (53.07)
Tobramycin	66/155 (42.58)	63/102 (61.76)	64/81 (79.01)	34/53 (64.15)		15/31 (48.39)	18/29 (62.07)	260/451 (57.65)
Trimethoprim/ Sulfamethoxazole		66/102 (64.71)	52/82 (63.41)	41/53 (77.36)	4/49 (8.16)	15/31 (48.39)	15/35 (42.86)	193/352 (54.83)

Data that were available in Microbiology Laboratory reports were informed. PA: *P. aeruginosa*; KP: *K. pneumoniae*; EC: *E. coli*; AB: *A. baumannii*; SM: *S. maltophilia*; ECl: *E. cloacae*; OGNB: Other GNB. OGNB include: *P. mirabilis* (*n* = 9). *S. marcescens* (*n* = 9), *Burkholderia* spp. (*n* = 9), *A. lwoffi* (*n* = 8), and *K. oxytoca* (*n* = 5).
